# Correction to: Regulation of Src tumor activity by its N-terminal intrinsically disordered region

**DOI:** 10.1038/s41388-022-02231-y

**Published:** 2022-02-21

**Authors:** Emilie Aponte, Marie Lafitte, Audrey Sirvent, Valérie Simon, Maud Barbery, Elise Fourgous, Yvan Boublik, Mariano Maffei, Florence Armand, Romain Hamelin, Julie Pannequin, Philippe Fort, Miquel Pons, Serge Roche

**Affiliations:** 1 CRBM, CNRS, Univ. Montpellier, Montpellier, France; 2 Equipe labellisée Ligue Contre le Cancer, CRBM, CNRS, Univ. Montpellier, Montpellier, France; 3 Biomolecular NMR laboratory, Department of Inorganic and Organic Chemistry, University of Barcelona, Barcelona, Spain; 4 Evvivax srl—Via di Castel Romano, Rome, Italy; 5 Proteomics Core Facility, School of Life Sciences, École Polytechnique Fédérale de Lausanne (EPFL), Lausanne, Switzerland; 6 IGF, CNRS, Univ. Montpellier, Montpellier, France

**Keywords:** Oncogenes, Growth factor signalling

Correction to: *Oncogene* 10.1038/s41388-021-02092-x, published online 9 Jan 2022

This article was inadvertently published with regular access instead of open access. The copyright holder was incorrectly given as The Author(s), under exclusive licence to Springer Nature Limited and the licence information was missing. The original article has been corrected.

The copyright of the article has changed on 25 January 2022 to © The Author(s) 2022 and the article is forthwith distributed under a Creative Commons Attribution 4.0 International License, which permits use, sharing, adaptation, distribution and reproduction in any medium or format, as long as you give appropriate credit to the original author(s) and the source, provide a link to the Creative Commons licence, and indicate if changes were made. The images or other third party material in this article are included in the article’s Creative Commons licence, unless indicated otherwise in a credit line to the material. If material is not included in the article’s Creative Commons licence and your intended use is not permitted by statutory regulation or exceeds the permitted use, you will need to obtain permission directly from the copyright holder. To view a copy of this licence, visit http://creativecommons.org/licenses/by/4.0/.

