# Supplementary material for: Regulation of Src tumor activity by its N-terminal intrinsically disordered region
Source: Oncogene. 2022 Jan 9;41(7):960–70. doi: 10.1038/s41388-021-02092-x (PMC8837538; doi:10.1038/s41388-021-02092-x)
Supplement: Supplementary file 1 — supplementary material [file 41388_2021_2092_MOESM1_ESM.pdf]

Supplementary information

Table S1. Label-free tyrosine phospho-proteomic analysis of Src-ULBR signaling in SW620 cells.

Figure S1. ULBR signaling in Src-transformed NIH3T3 cells. a: ULBR regulates SFK activity and protein tyrosine phosphorylation (n=3). **b**: ULBR regulates p38 MAPK and Stat3 signaling (n=3). **c:** ULBR does not affect Src-induced dissolution of actin fibers. Immunoblots and relative band intensity quantification of whole-cell lysates is indicated. A representative example of actin immunostaining (left) and the quantification (right) of cells transformed with indicated Src mutants showing remaining actin fibers (mean ± SD; n=3); ns: p>0.05; *p<0.05; **p<0.01; ***p<0.001; Student's *t* test.

Figure S2. Quantification of ULBR inactivation on Src activity and signaling. a: quantification normalized to the Src YF condition set a 100% in 3T3 cells stably transduced with the indicated constructs. **b:** quantification normalized to the Src WT condition set a 100% in HEK293T cells transfected with indicated Src constructs. **c:** quantification normalized to the Src condition set a 100% in SW620 cells stably transduced with indicated constructs (mean ± SD; a: n=3; b: n=5; c: n=5). ns: p>0.05; *p<0.05; **p<0.01; ***p<0.001; Student's *t* test.

Figure S3. Quantification of Src co-localization with Src-ULBR mutants in HEK293T cells. a: modular structure of Src constructs transfected in HEK293T cells. **b**: % of transfected cells that exhibit co-localization between Src-mCherry and indicated Src-GFP constructs from >20 transfected cells analyzed per condition (mean ± SD; n=3). **c**: Representative confocal image of direct fluorescence of HEK293T cells expressing Src-GFP or indicated Src-GFP ULBR mutants alone from >20 transfected cells analyzed.

Figure S4. ULBR does not regulates Src kinase activity in vitro. Western blotting (**a**) and quantification (**b**) showing the time course and concentration-dependence of Enolase phosphorylation by immunopurified Src-GFP and Src3A-GFP using an in vitro kinase assay described in methods (n=2).

Figure S5. ULBR modulates Src-induced RTK and Fyn tyrosine phosphorylation. a: ULBR-dependent Src signaling in SW620 cells. **b**: phospho-RTKs (left) and phospho-kinase antibody array analysis (right) using SW620 cells expressing indicated constructs. Specific Src-ULBR substrates are highlighted. **c**: quantification of pRTK (left) and phospho-kinase array (right) relative to the mock condition. Representative example from 2 independent experiments.

Figure S6. Fyn and EPHA2 are important mediators of ULBR-Src signaling in SW620 cancer cells. a: cell invasion of SW620 expressing or not Src or Src3A mutant and transfected with indicated siRNA (mean ± SD; n= 3). **b**: Src does not affect Fyn mRNA level in SW620 cells. Is shown the relative mRNA Fyn level in SW620 cells expressing indicated Src construct and transfected with indicated siRNA (mean ± SD; n= 3). **c**: EPHA2 protein level in SW620 cells expressing indicated Src construct and transfected with indicated siRNA (n=2). **d:** quantification of Src-induced MAPK activation (normalized to the Src WT conditions set at 100%) in SW620 infected with indicated virus and transfected with indicated siRNA (mean ± SD; n=3). **e**: ULBR-dependent interaction between Src and Fyn signaling in HEK293T cells: a representative example (top) and the quantification of MAPK activity (bottom) (mean ± SD; n=3); ns: $p>0.05$; *$p<0.05$; **$p<0.01$; ***$p<0.001$; Student's *t* test.

Figure S1

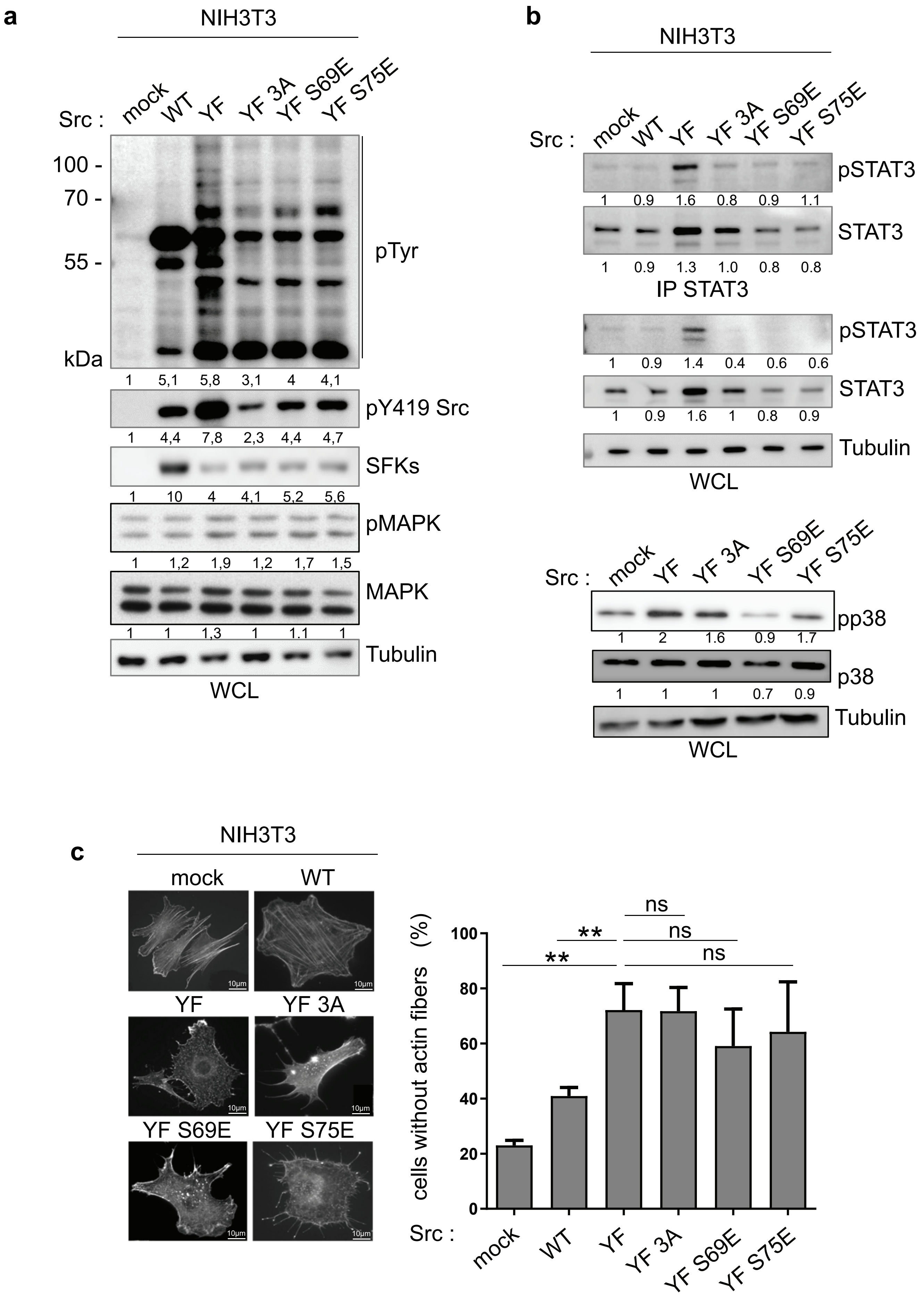
a
NIH3T3
Src :
mock
WT
YF
YF 3A
YF S69E
YF S75E
100 -
70 -
55 -
kDa
pTyr
1 5,1 5,8 3,1 4 4,1
pY419 Src
1 4,4 7,8 2,3 4,4 4,7
SFKs
1 10 4 4,1 5,2 5,6
pMAPK
1 1,2 1,9 1,2 1,7 1,5
MAPK
1 1 1,3 1 1.1 1
Tubulin
WCL
b
NIH3T3
Src :
mock
WT
YF
YF 3A
YF S69E
YF S75E
pSTAT3
1 0.9 1.6 0.8 0.9 1.1
STAT3
1 0.9 1.3 1.0 0.8 0.8
IP STAT3
pSTAT3
1 0.9 1.4 0.4 0.6 0.6
STAT3
1 0.9 1.6 1 0.8 0.9
Tubulin
WCL
Src :
mock
YF
YF 3A
YF S69E
YF S75E
pp38
1 2 1.6 0.9 1.7
p38
1 1 1 0.7 0.9
Tubulin
WCL
c
NIH3T3
mock
WT
YF
YF 3A
YF S69E
YF S75E
10µm
cells without actin fibers (%)
100
80
60
40
20
0
ns
**
ns
**
ns
Src :
mock
WT
YF
YF 3A
YF S69E
YF S75E

Figure S2

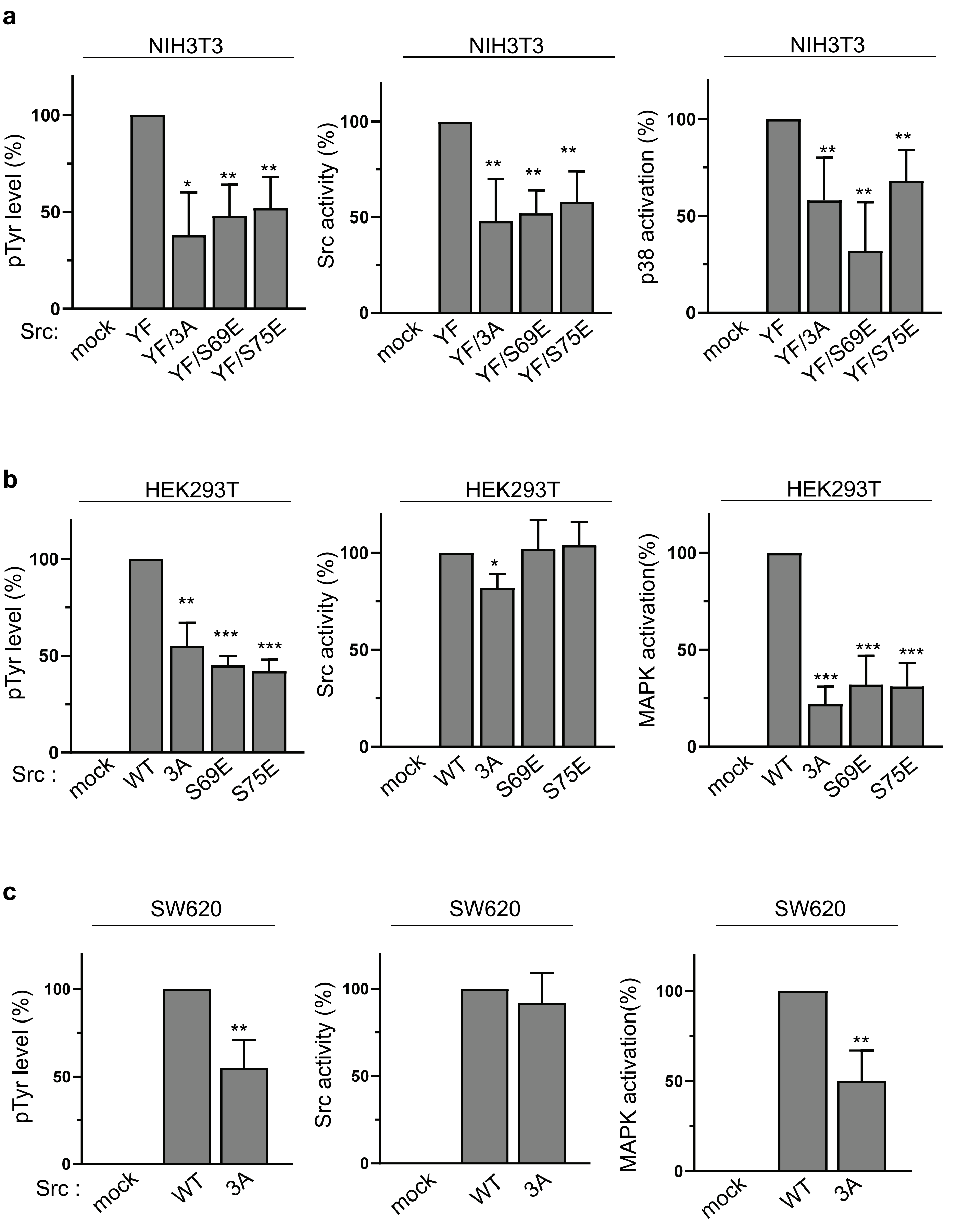
a
NIH3T3
pTyr level (%)
Src activity (%)
p38 activation (%)
Src:
mock
YF
YF/3A
YF/S69E
YF/S75E
b
HEK293T
pTyr level (%)
Src activity (%)
MAPK activation(%)
Src :
mock
WT
3A
S69E
S75E
c
SW620
pTyr level (%)
Src activity (%)
MAPK activation(%)
Src :
mock
WT
3A

Figure S3

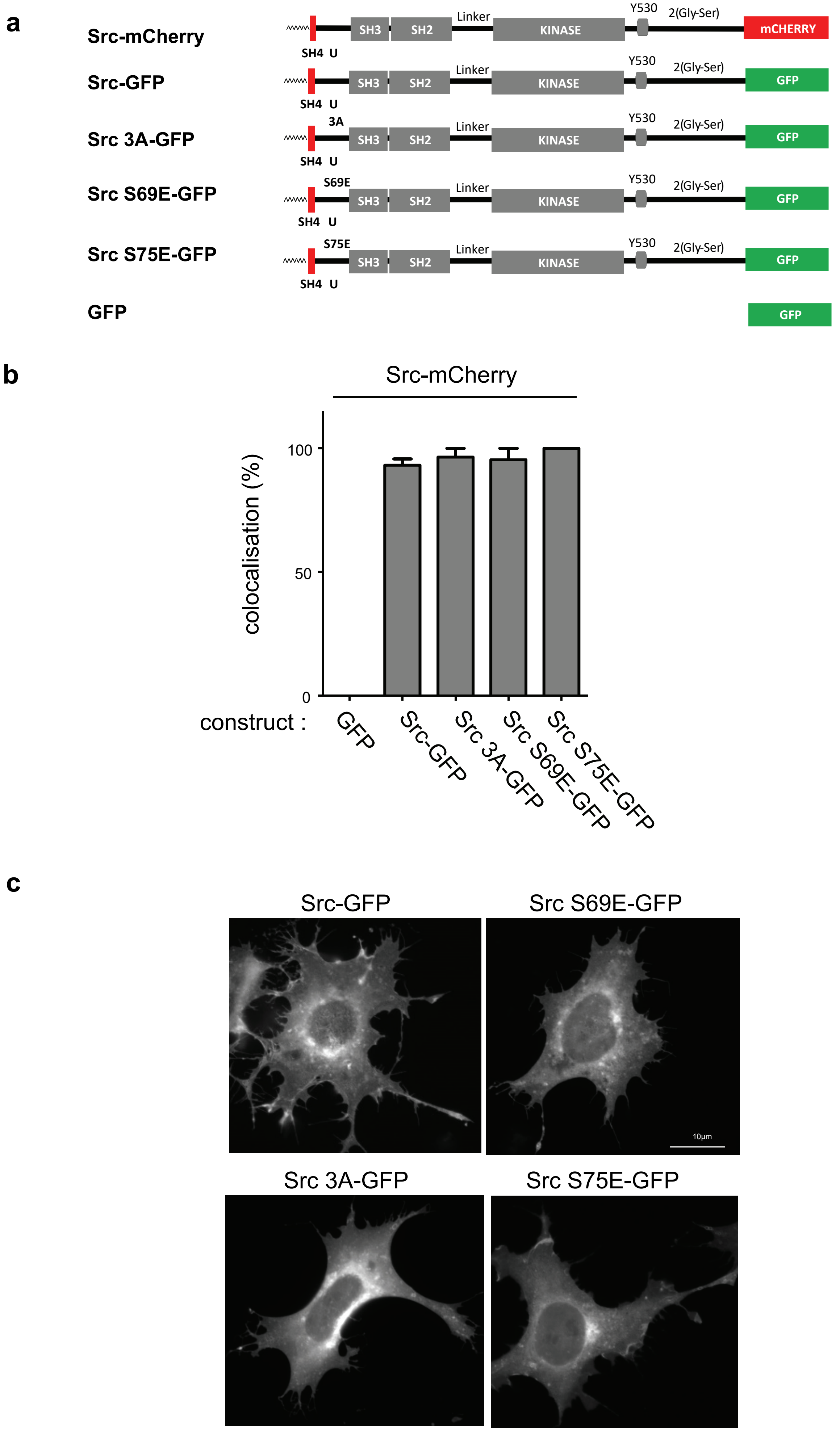
a
Src-mCherry
Src-GFP
Src 3A-GFP
Src S69E-GFP
Src S75E-GFP
GFP
SH4 U
3A
S69E
S75E
SH3
SH2
Linker
KINASE
Y530
2(Gly-Ser)
mCHERRY
GFP
b
Src-mCherry
colocalisation (%)
100
50
0
construct :
GFP
Src-GFP
Src 3A-GFP
Src S69E-GFP
Src S75E-GFP
c
Src-GFP
Src S69E-GFP
10µm
Src 3A-GFP
Src S75E-GFP

Figure S4

a

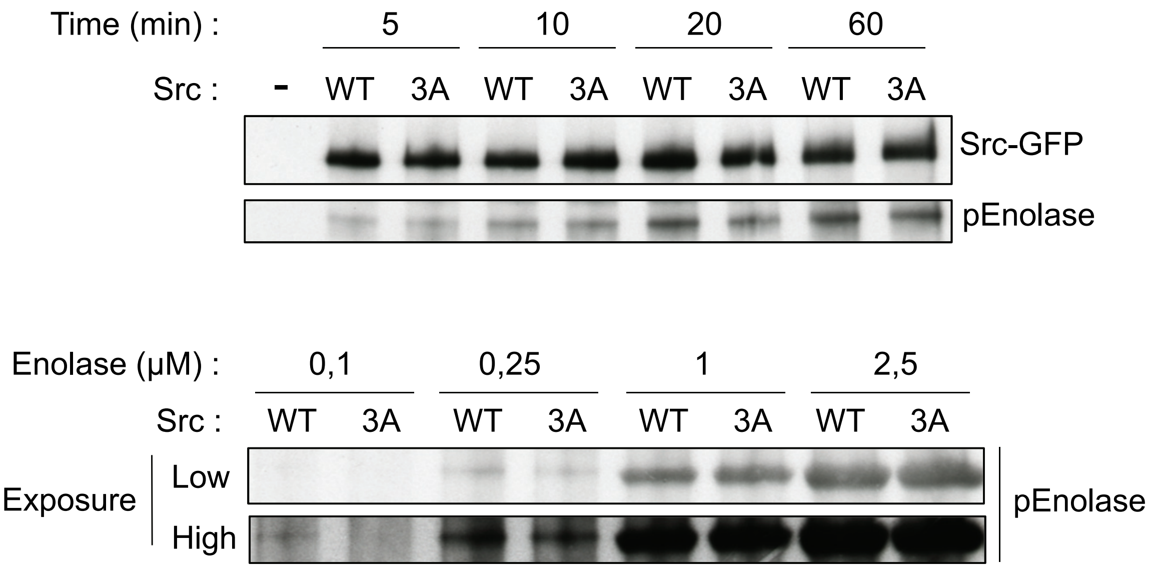
Time (min) :
5
10
20
60
Src :
-
WT
3A
WT
3A
WT
3A
WT
3A
WT
3A
Src-GFP
pEnolase
Enolase (μM) :
0,1
0,25
1
2,5
Src :
WT
3A
WT
3A
WT
3A
WT
3A
Exposure
Low
High
pEnolase

b

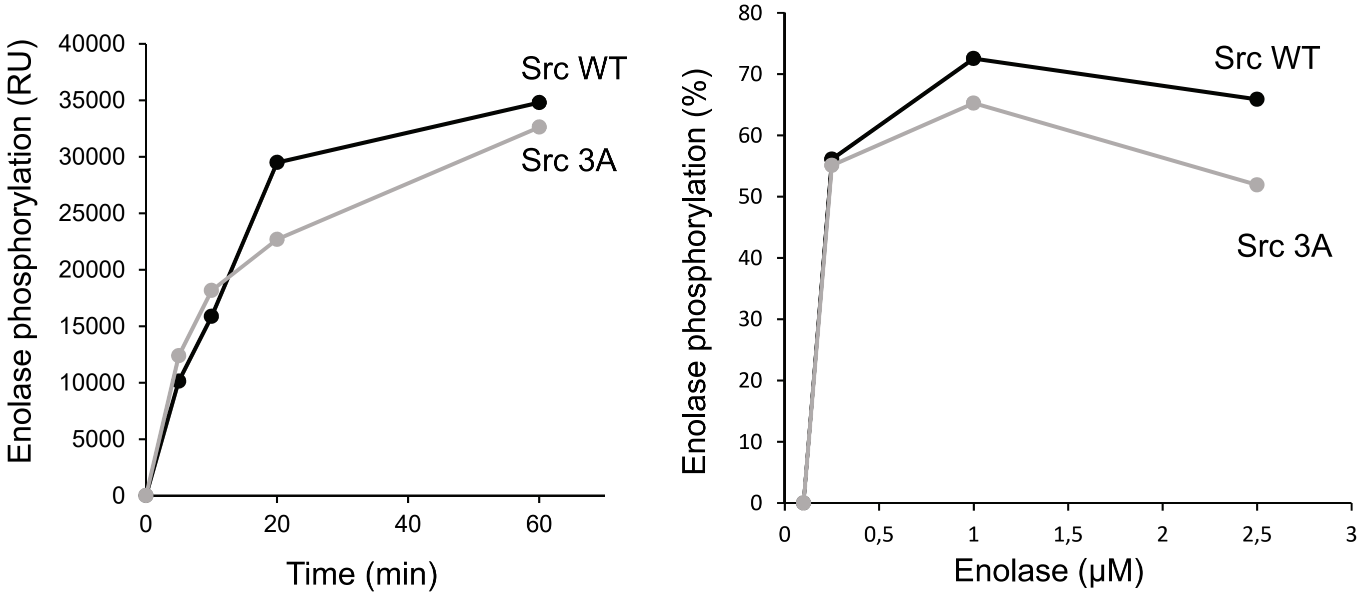
Enolase phosphorylation (RU)
40000
35000
30000
25000
20000
15000
10000
5000
0
0
20
40
60
Time (min)
Src WT
Src 3A
Enolase phosphorylation (%)
80
70
60
50
40
30
20
10
0
0
0,5
1
1,5
2
2,5
3
Enolase (μM)
Src WT
Src 3A

Figure S5

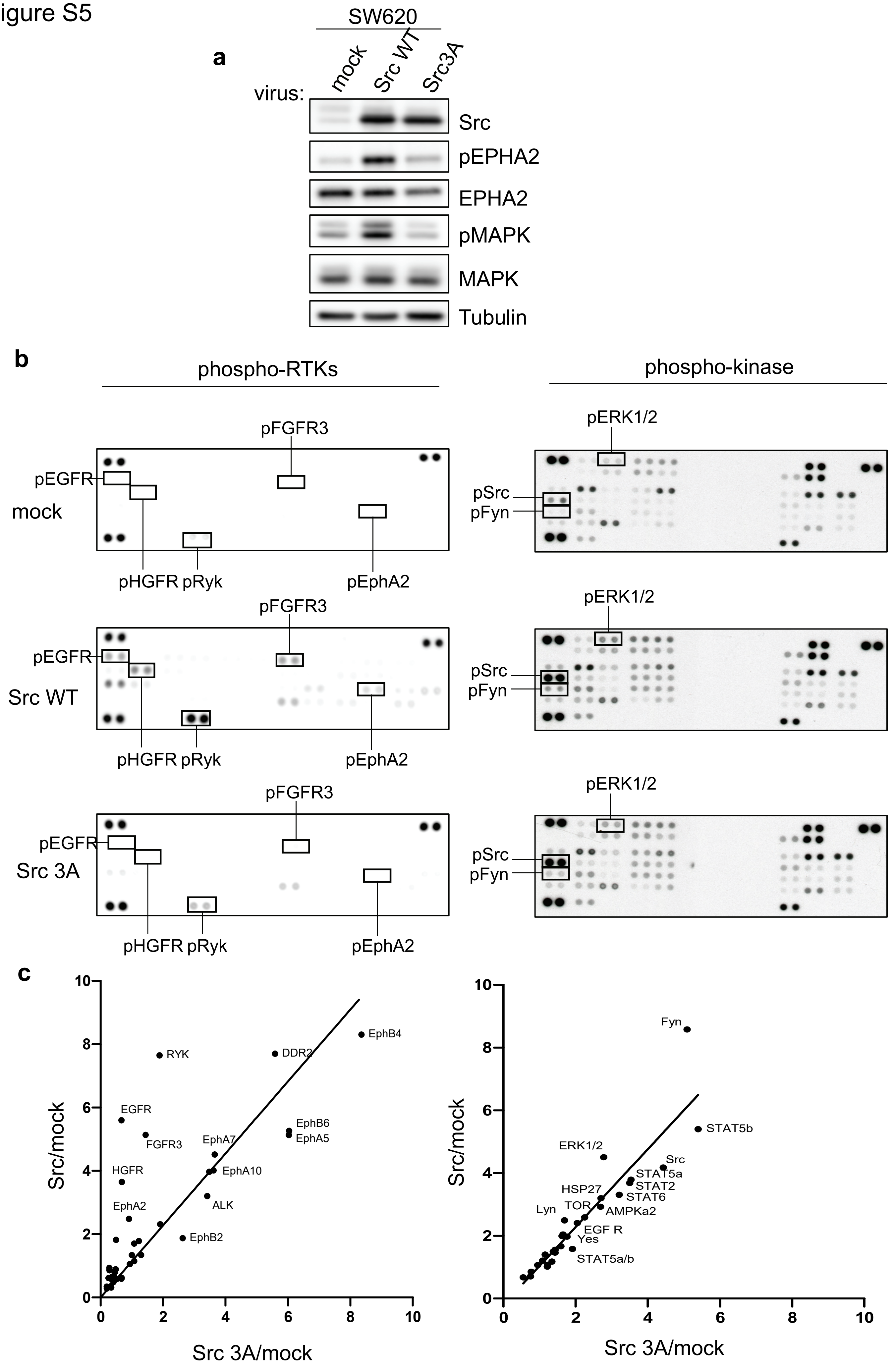

a
SW620
virus:
mock
Src WT
Src3A
Src
pEPHA2
EPHA2
pMAPK
MAPK
Tubulin
b
phospho-RTKs
phospho-kinase
mock
Src WT
Src 3A
pEGFR
pFGFR3
pHGFR
pRyk
pEphA2
pERK1/2
pSrc
pFyn
c
Src/mock
Src 3A/mock
RYK
DDR2
EphB4
EGFR
FGFR3
EphB6
EphA5
EphA7
HGFR
EphA10
ALK
EphA2
EphB2
Fyn
STAT5b
ERK1/2
Src
STAT5a
STAT2
STAT6
HSP27
TOR
AMPKa2
Lyn
EGF R
Yes
STAT5a/b
0
2
4
6
8
10

Figure S6

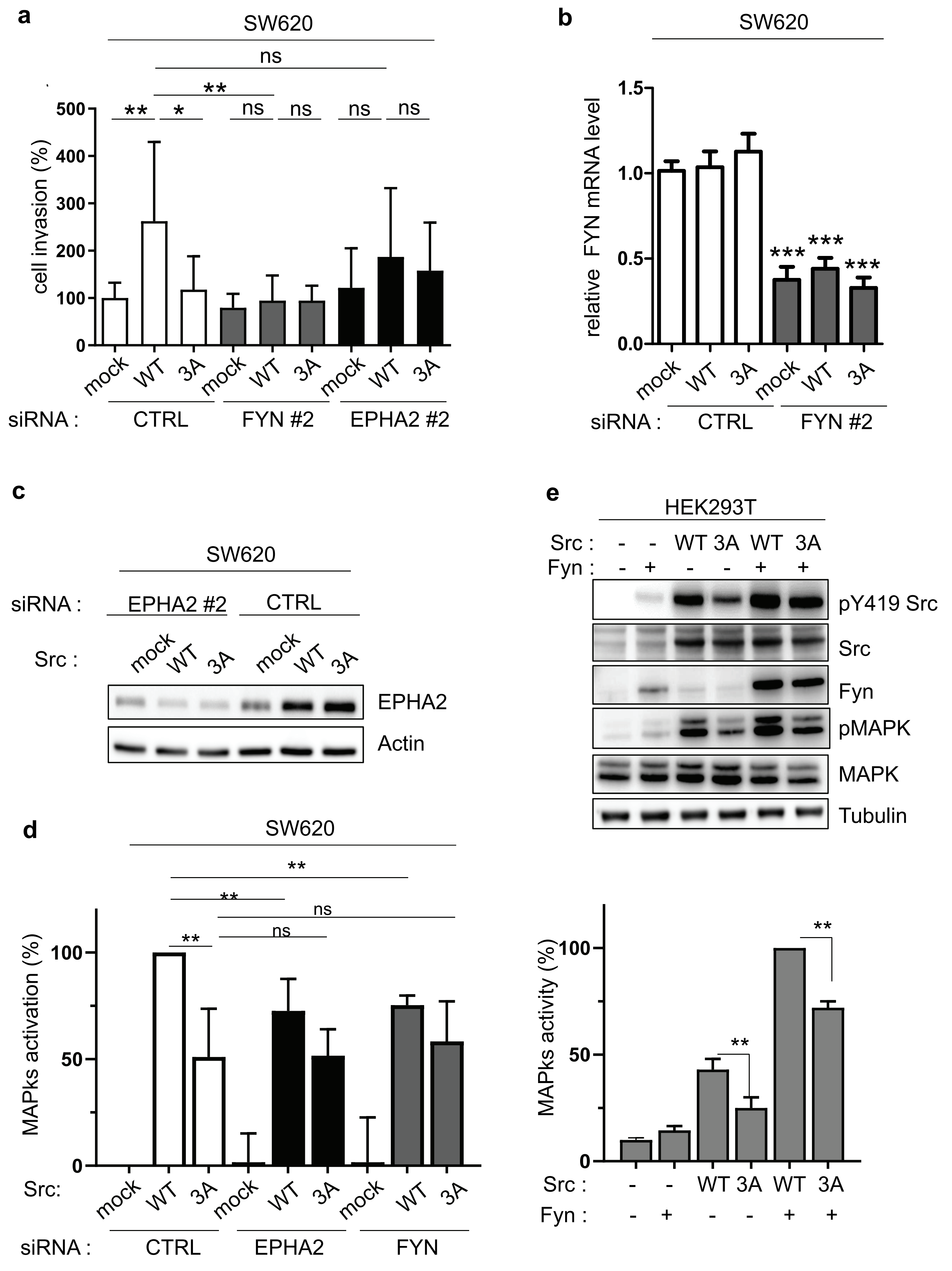
a
SW620
ns
**
** * ns ns ns ns
cell invasion (%)
500
400
300
200
100
0
mock WT 3A mock WT 3A mock WT 3A
siRNA : CTRL FYN #2 EPHA2 #2
b
SW620
relative FYN mRNA level
1.5
1.0
0.5
0.0
*** *** ***
mock WT 3A mock WT 3A
siRNA : CTRL FYN #2
c
SW620
siRNA : EPHA2 #2 CTRL
Src : mock WT 3A mock WT 3A
EPHA2
Actin
d
SW620
**
**
ns
ns
**
MAPKs activation (%)
100
50
0
Src: mock WT 3A mock WT 3A mock WT 3A
siRNA : CTRL EPHA2 FYN
e
HEK293T
Src : - - WT 3A WT 3A
Fyn : - + - - + +
pY419 Src
Src
Fyn
pMAPK
MAPK
Tubulin
MAPKs activity (%)
100
50
0
**
**
Src : - - WT 3A WT 3A
Fyn : - + - - + +
